# Large-Scale Place Recognition Based on Camera-LiDAR Fused Descriptor

**DOI:** 10.3390/s20102870

**Published:** 2020-05-19

**Authors:** Shaorong Xie, Chao Pan, Yaxin Peng, Ke Liu, Shihui Ying

**Affiliations:** 1 School of Computer Engineering and Science, Shanghai University, Shanghai 200444, China; srxie@shu.edu.cn; 2 School of Mechatronic Engineering and Automation, Shanghai University, Shanghai 200444, China; 767937587@shu.edu.cn; 3 Department of Mathematics, School of Science, Shanghai University, Shanghai 200444, China; liuke6@i.shu.edu.cn (K.L.); shying@shu.edu.cn (S.Y.)

**Keywords:** place recognition, retrieval, sensor fusion, deep learning

## Abstract

In the field of autonomous driving, carriers are equipped with a variety of sensors, including cameras and LiDARs. However, the camera suffers from problems of illumination and occlusion, and the LiDAR encounters motion distortion, degenerate environment and limited ranging distance. Therefore, fusing the information from these two sensors deserves to be explored. In this paper, we propose a fusion network which robustly captures both the image and point cloud descriptors to solve the place recognition problem. Our contribution can be summarized as: (1) applying the trimmed strategy in the point cloud global feature aggregation to improve the recognition performance, (2) building a compact fusion framework which captures both the robust representation of the image and 3D point cloud, and (3) learning a proper metric to describe the similarity of our fused global feature. The experiments on KITTI and KAIST datasets show that the proposed fused descriptor is more robust and discriminative than the single sensor descriptor.

## 1. Introduction

Place recognition has received a significant amount of attention in various fields including computer vision [1,2,3,4,5,6], autonomous driving systems [7,8,9,10] and augmented reality [11]. In these tasks, place recognition addresses a question of “where am I in a route”. This question is related to how to recognize places based on their appearance and surrounding structure. Therefore, this technology plays an important role in the Simultaneous Localization And Mapping (SLAM) [12] for the robotic and autonomous driving system.

Given a place recognition problem, a common and direct method is to find the most matching candidate place(s) of the current place, which is stored in a database of the environment map [13,14]. Its fundamental scientific question is to determine the appropriate representation (of a place) to distinguish similar and dissimilar places. Traditionally, features for visual information, such as Oriented FAST and Rotated Brief (ORB) [15], Scale-Invariant Feature Transform (SIFT) [16] and Speeded Up Robust Features (SURF) [17], have been proposed to describe the strong corner features in each image. Moreover, some methods like the Bag-of-Words (BoW) [18,19], Vector of Locally Aggregated Descriptors (VLAD) [20] and Fisher vector [21] have been used to aggregate these local features into a single vector representation for the entire image representing a certain place. Existing well-performing image retrieval [22,23,24,25] and place recognition [6,26] systems mainly utilize traditional hand-crafted features or processing models, which might fail to build feature representation appropriately, since hand-crafted feature representation perhaps only extract limited features.

Recently, with massive data and the development of powerful hardware, data-driven methods have emerged. Deep neural network (DNN) is the most representative method, which has a good capability to extract features, showing strong advantages in the field of computer vision and pattern recognition. The success of deep learning has been particularly noticeable on 2D images where convolution kernels can be easily applied to the regular 2D lattice grid structure of the image. Besides, these kernels can also aggregate the information of surrounding pixels to the following layer. Meanwhile, convolutional neural network (CNN) ResNet [27] and VGGNet [28] have been widely used in the image retrieval and detection field. For visual information, all kinds of networks are designed to extract visual descriptor or conduct image retrieval directly [29,30,31,32,33,34]. Reference [29] is the first work to introduce a CNN-based place recognition system, and Reference [30] provides a thorough investigation of the utility and viewpoint-invariant properties of deep learned features for the place recognition. After them, more and more CNN-based place recognition methods [31,32,33] have been proposed. NetVLAD [34], a generalized VLAD layer, combines the advantages of the classical VLAD and data-driven methods. Based on the NetVLAD, the GhostVLAD [35] proposes a variant that added ghost clusters. The network automatically learns how to distinguish the low quality images and then gives them lower weights, which helps to generate a better template. Generally speaking, these CNN-based recognition approaches have been proposed rapidly and most of them perform better than those traditional approaches.

Intuitively, the sun exposure conditions and things in the scene may continuously change. Even the images acquired in the same place often look different. It is well known that these visual-based methods will encounter problems of illumination, occlusion and the change of viewpoint. To avoid these disadvantages of visual sensors, some 3D point clouds based place recognition methods, captured by LiDAR (Light Detection And Ranging), are introduced [36]. Unfortunately, it is more challenging for convolution kernels to work on orderless 3D point clouds. Compared with the visual methods, there are indeed less methods to deal with the point cloud, no matter classical or data-driven methods, since the input 3D information is orderless and sparse in $R^{3}$ [37].

For a 3D point cloud, the 3D shape context [38] descriptor is used to obtain local 3D features. Moreover, Point Feature Histogram [39] and Signature Histogram of Orientation (SHOT) [40] generate histograms based on geometric attribution. However, these methods consume much time and computing resources. By contrast, Fast Point Feature Histogram (FPFH) [41,42], 3D SIFT [43] can extract local point cloud features with low cost in computation, but they do not perform well at sparse space locations. Some global descriptor extraction methods such as Ensemble of Shape Functions (ESF) [44], Normal Aligned Radial Feature (NARF) [45] and Viewpoint Feature Histogram (VFH) [46] are able to ignore sparse distribution of point cloud at some local locations.

Several deep networks attempted to cope with this challenge by transforming the input point clouds into regular 3D volumetric representations. For instance, the 3D ShapeNet [47], volumetric CNN [48] and Vote3Deep [49] resolve 3D object detection, classification and recognition, respectively. The recent PointNet [50], PointNet++ [51] GVCNN [52] and PVNet [53] made it possible to directly input the original point cloud into network. However, they were designed to handle small object classifications and indoor scene segmentations. In [36], the PointNetVLAD, which is the combination of PointNet and NetVLAD, utilizes the unique VLAD layer to aggregate LiDAR features extracted by the PointNet [50], which makes the feature extraction more effective. However, point clouds may encounter limited ranging distance, and also suffer from motion distortion and some disturbances.

**Figure 1:**
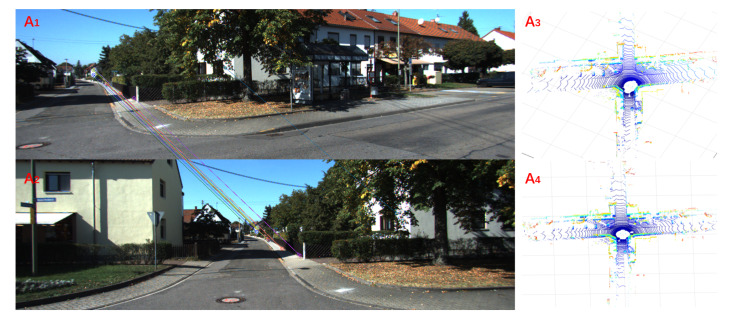
Complementarity between 3D point clouds and images. $A_{1}$ and $A_{2}$ are the images of the same place, which look very different since they are from two different views. $A_{3}$ and $A_{4}$ are the corresponding point clouds of images $A_{1}$ and $A_{2}$.

**Figure 2:**
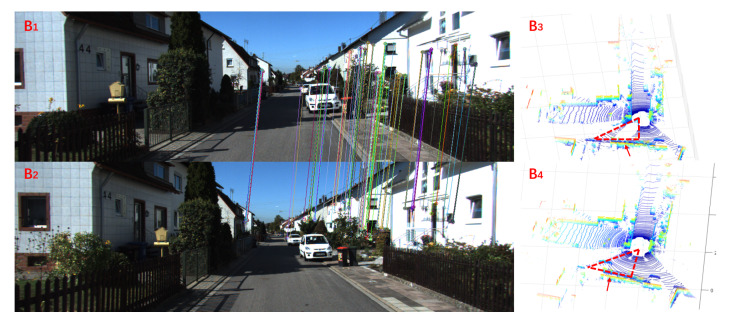
Complementarity between images and 3D point clouds. $B_{1}$ and $B_{2}$ are two images at the same place, which is supplementary information to their corresponding point clouds $B_{3}$ and $B_{4}$. $B_{3}$ and $B_{4}$ look very different (marked by red triangular regions).

Because of the complexity and variability of outdoor large-scale places, it may not be effective for place recognition by only using the image or point feature. In fact, only using a single sensor will have limitations in a specific scenario. For example, in Figure 1, $A_{1}$ and $A_{2}$ represent images for the same place from two different views, $A_{3}$ represents the point cloud corresponding to $A_{1}$, while $A_{4}$ represents that of $A_{2}$. These two images captured in the different viewpoints only have a few matching pairs. As a result, it is hard to find out the relationship between these two images only using the visual information. Meanwhile, their point clouds are almost the same, meaning that the LiDAR information is helpful for obtaining a proper conclusion. As depicted in Figure 2, the LiDAR features $B_{3}$ and $B_{4}$ are different (marked by red triangular regions), but their visual data $B_{1}$ and $B_{2}$ are sufficient to prove these two frames are captured in the same place. Actually, these two cases show that there exists complementarity between the image and point cloud. Therefore, we hope to fuse the information of image and point cloud, since both of them possess their own peculiarities. Some pioneers have tried to fuse these two kinds of input by establishing the relationship between the rectilinear feature in image and the planar intersection in point cloud [54] or combining them in the final decision [55]. Unlike the above methods, our method uses networks to learn the features fusing two kinds of input, which has a more tight coupling.

In this paper we propose a camera-LiDAR sensors fusion method to extract fused global descriptors for place recognition via a deep neural network. The contributions of our work are summarized as—(1) applying the trimmed strategy for non-informative clusters to reduce the impact of some unrepresentative information in a 3D point cloud; (2) proposing a fusion neural network concatenating both the visual descriptor and 3D spatial global descriptor; and (3) using metric learning ideology to reduce the distance of fused descriptors among similar places and extend it among dissimilar places. With a metric learning loss, the whole network is optimized to get an appropriate mapping from the 3D Euclidean space to the descriptor space, in which the descriptor can distinguish the different places easier.

This paper is organized as follows. Section 2 introduces some methods directly related to the approach in this paper. In Section 3 we introduce the framework of our neural network based method that can generate global fusion descriptor based on visual and 3D point information. Section 4 presents the datasets and experimental results. The paper is concluded in Section 5.

## 2. Related Works

### 2.1. Visual Feature Extractor

**Figure 3:**
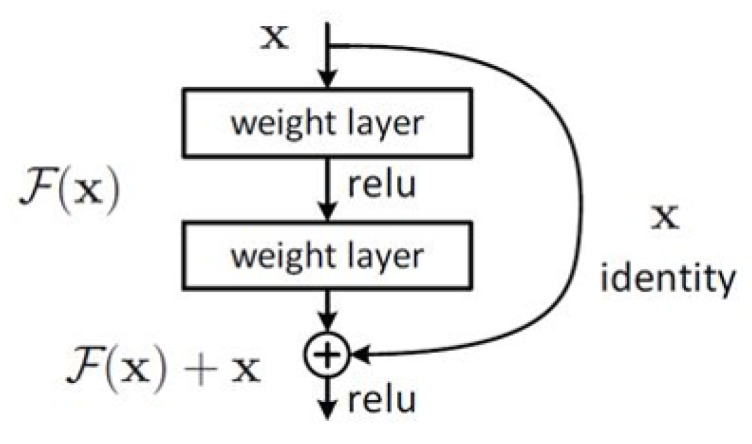
Residual block in ResNet [27].

ResNet [27] is the basis of our proposed approach for extracting image features. It performs well in image recognition, and solves the degeneration problem when training deep neural networks. This method presents a residual learning framework to ease the training of networks that are substantially deeper than those used previously. A principal module named residual block is shown in Figure 3.

This method shows that deep residual learning can solve the degeneration problem. For a stacked layers structure, *X* is the input of the first layer of the residual block. Formally, denoting the desired output as H(X), the residual block learns another nonlinear mapping F(X)=H(X)−X to get the residual value. It not only solves the deep learning degeneration problem, but also apparently reduces the computational complexity. With the accumulation of residual block, the ResNet is formed naturally to obtain the deeper features of images.

### 2.2. Global Descriptor of 3D Point Cloud via Netvlad

**Figure 4:**
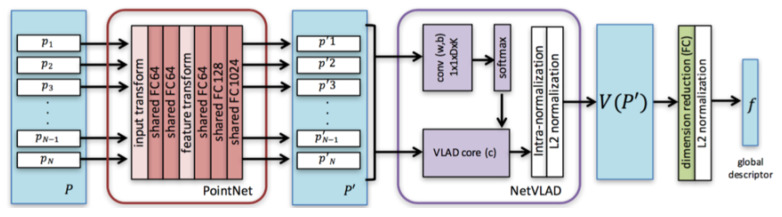
The framework of PointNetVLAD [36].

VLAD [20], as mentioned before, is a classical hand-crafted feature extractor method which can aggregate these local features into a single vector representation for the entire image representing a certain place. Fortunately NetVLAD [34] can combine the advantages of VLAD and data-driven methods. Based on PointNet [50] and NetVLAD, PointNetVLAD [36] focuses on the place recognition in scene retrieval, and outperforms the maxpool layer features extracted from the original PointNet. Its framework is shown in Figure 4.

The main idea of PointNetVLAD is to view the point features of every point cloud learned from the PointNet block as the input of NetVLAD block, and then utilize the input *N D*-dimensional feature points to aggregate a single (K·D)×1 global descriptor vector as

(1) $$V_{k}=\sum_{i=1}^{N}\frac{e^{w_{k}\cdot p_{i}^{\prime }+b_{k}}}{\sum_{k^{\prime }}e^{w_{k^{\prime }}\cdot p_{i}^{\prime }+b_{k^{\prime }}}}\left(p_{i}^{\prime }-c_{k}\right),$$

where the cluster centers $\left\{c_{k}\right\}$ and parameters $\left\{w_{k}\right\},\left\{b_{k}\right\}$ are trainable from the network. One branch of the NetVLAD block computes the difference between local features and cluster centers, while the softmax part determines the weights of the points to each cluster centers.

The advantage of PointNetVLAD is the acquirement of the global descriptor for the whole point cloud, which has less redundant feature comparing with those local feature extraction operators. However, the global descriptor may be disturbed by some outliers and isolated points. We will avoid these problems via our modified local-to-global fusion descriptor retrieval.

## 3. Proposed Method

We list here some notations in our algorithm. For each scene, the source data $\left(I,\hat{P}\right)$ include the image information *I* and the corresponding 3D point cloud $\hat{P}$. Note that $\hat{P}$, acquired from LiDAR, may have different sizes. For feature extraction facility in deep neural network, we apply a down-sampling preprocess to 3D source point cloud $\hat{P}$, to get the fixed size point cloud $P={\left\{p_{l}|p_{l}\in R^{3}\right\}}_{l=1}^{N}$ before feature extraction, that is, $\hat{P}\overset{sampling}{⟶}P={\left\{p_{l}|p_{l}\in R^{3}\right\}}_{l=1}^{N}$.

**Figure 5:**
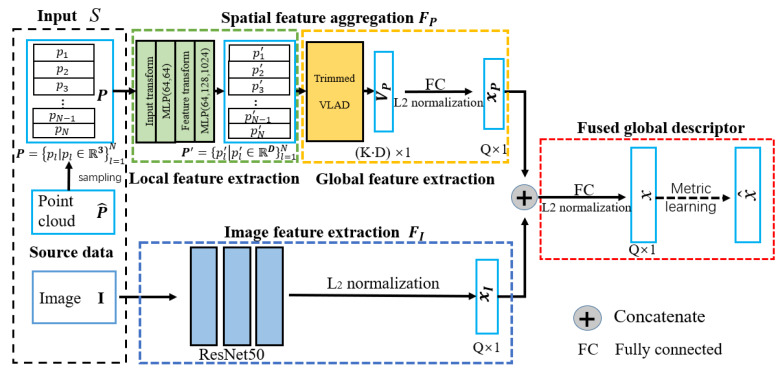
Network architecture.

We then use the neural network to generate a robust and compact representation for a specific place (I,P). For acquiring this representation, we need to learn a proper mapping F(·) from the input data space S={(I,P)} to a new space for facilitating the place retrieval. The whole framework is shown in Figure 5. A good mapping should consist of two modules—the efficient feature extraction operator F (the green, yellow and blue blocks in Figure 5) and the best metric M to evaluate of similarity of feature descriptors (the red block in Figure 5). This process can be formulated with

(2) $$\begin{array}{ccc} S & \overset{F(\cdot )}{⟶}X & \overset{M(\cdot )}{⟶}\hat{X}\\ S & \mapsto x=F(S) & \mapsto \hat{x}=M\circ F\left(S\right) \end{array}$$

for a certain place S=(I,P).

Firstly, we should build a feature extraction operator F to represent the place accurately and robustly. Note that each place has the image and the corresponding 3D point cloud. So, the feature extraction operator F has two map branches $F_{I}$ and $F_{P}$ to extract visual feature $x_{I}$ and 3D point cloud feature $x_{P}$ respectively, that is, $F=F_{I}⊕F_{P}$. In this part, the input data of camera and LiDAR information, according to their data patterns, are fed into image feature extraction branch (the blue block) and 3D Spatial feature aggregation branch (the green and yellow blocks), and get the respective descriptors $x_{I}$ and $x_{P}$. Specifically, we introduce a trimmed clustering method to extract the global descriptor $x_{P}$ for 3D point cloud via ignoring the non-informative point feature clusters.

To avoid the shortcoming of both visual and LiDAR information process, a concatenating and fully connection operation are applied to build the global fusion feature, that is, $x=x_{I}⊕x_{P}$. Furthermore, the triplet constraints of the sampling are used to learn the best metric M for finding a good viewpoint to describe the similarity of sampling. Finally, the model parameters are optimized to get an appropriate mapping relationship between the input data and the fused global descriptor $\hat{x}$ via an end-to-end training. We will introduce these modules in the following section.

### 3.1. Spatial Feature Aggregation with Trimmed Strategy

In this part, we apply a local-to-global methodology to aggregate the point cloud features. This procedure begins with a local feature extraction (the green block), then follows a global feature extraction (the yellow block) for point cloud.

#### 3.1.1. Local Feature Extraction for Point Cloud

For the input point cloud *P*, the Multi-Layer Perception (MLP) and feature transform are applied to extract the local spatial feature information for point cloud by mapping each 3D dimensional point $p_{l}\in R^{3}$ into a higher dimensional space $p_{l}^{\prime }\in R^{D}$, for D≫3. That is,

$$P={\left\{p_{l}|p_{l}\in R^{3}\right\}}_{l=1}^{N}⟶P^{\prime }={\left\{p_{l}^{\prime }|p_{l}^{\prime }\in R^{D}\right\}}_{l=1}^{N}.$$

This part of the network (the green block) extracts some local rotation invariant spatial features for each single point $p_{l}\in R^{3}$ from the input point cloud *P*. Thus, the original 3D point is mapped into a higher dimensional local feature vector. This redundant representation makes the points maintain much more their own information, which is widely used as the feature extraction module for point cloud processing, such as PointNet [50]. Some redundant information will be omitted to form a compact global descriptor in the following process.

#### 3.1.2. Global Feature Extraction with Trimmed Clustering

**Figure 6:**
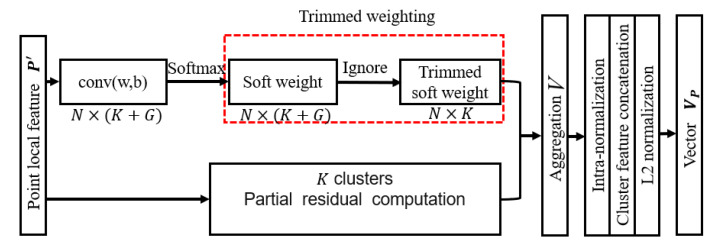
Diagram of the trimmed Vector of Locally Aggregated Descriptors (VLAD).

Comparing with PointNetVLAD [36], we introduce a novel trimmed VLAD block for point clouds in this module (the yellow block in Figure 5). Originally, the *K* cluster centers $K={\left\{c_{k}|c_{k}\in R^{D}\right\}}_{k=1}^{K}$ and their corresponding weights $W_{k}\left(\cdot \right)=W_{k}\left(w_{k},b_{k}\right)\left(\cdot \right),k=1,\cdots ,K$ are learned to represent *K* special and prominent parts of the current 3D space, so that the outputs related to these cluster centers are distinctive. However, this procedure is sensitive to the complex environments captured by a 3D LiDAR. For avoiding this redundant information and environment disturbance robustly, we will introduce a trimmed strategy via ignoring the non-informative clustering of 3D point cloud, that is, the trimmed VLAD block. The details of this new block are shown in Figure 6.

Notice that not all the points and clusters are useful for forming a compact and robust representation of point cloud. For instance, some outliers or isolated points do not correctly reflect the features of point cloud $P^{\prime }$, so they are not helpful to the representation of $P^{\prime }$. Moreover, points belonging to this kind of dynamic clusters in a scene may exert a negative influence on the recognition precision. Under this consideration, we assume that there exist more cluster centers $G={\left\{c_{k}|c_{k}\in R^{D}\right\}}_{k=K+1}^{G}$ and its corresponding weights $W_{k}\left(\cdot \right)=W_{k}\left(w_{k},b_{k}\right)\left(\cdot \right),k=K+1,\cdots ,K+G$ to non-informative clusters for separating their influences.

For the given proper cluster size *K* and the non-informative cluster size *G*, the trimmed weights of an input descriptors $P^{\prime }={\left\{p_{l}^{\prime }|p_{l}^{\prime }\in R^{D}\right\}}_{l=1}^{N}$ can be computed via

(3) $$W_{k}\left(p_{l}^{\prime }\right)=\frac{e^{w_{k}\cdot p_{l}^{\prime }+b_{k}}}{\sum_{m\leq K+G}e^{w_{m}\cdot p_{l}^{\prime }+b_{m}}},\;\;\;\;\mathrm{for}\;k=1,\cdots ,K,$$

where $\left\{w_{k}\right\},\left\{b_{k}\right\},k=1,\cdots ,K$ are the weight parameters for proper clusters, and $\left\{w_{k}\right\},\left\{b_{k}\right\},k=K+1,\cdots ,G$ are the weight parameters for non-informative clusters. All of them are trainable via the trimmed weights sub-block (the red block in Figure 5). Note that the weight parameters for non-informative clusters are included into the summation in the denominator of Equation (3), which makes these non-informative clusters contribute to the soft assignments in the same manner as the original clusters K. Moreover, only the trimmed weights for the meaningful clusters K are computed, since the weights of the non-informative clusters will be ignored in the aggregation process.

For an input local descriptor $P^{\prime }={\left\{p_{l}^{\prime }|p_{l}^{\prime }\in R^{D}\right\}}_{l=1}^{N}$, the output is a K×D-dimension matrix $V=[V_{1},\cdots ,V_{K}]$ after the partial aggregation, in which $V_{k}$ can be written as

(4) $$V_{k}=\sum_{l=1}^{N}W_{k}\left(p_{l}^{\prime }\right)\left(p_{l}^{\prime }-c_{k}\right),\;\;\;\;\mathrm{for}\;k=1,\cdots ,K,$$

where $c_{k}$ is the proper cluster center, $W_{k}\left(p_{l}^{\prime }\right)$ is the trimmed weight of the local descriptor $p_{l}^{\prime }$ for the meaningful cluster K, and $p_{l}^{\prime }-c_{k}$ represents the difference between the local descriptor of sample and its corresponding meaningful cluster center $c_{k}$. The cluster centers and the differences (partial residual ) are computed in a single branch network. Then we assign the trimmed weight to partial residuals of the meaningful clusters in partial aggregation process for getting a global descriptor *V*. This trimmed-weight based aggregation process is robust for complex LiDAR data, since the non-informative clusters are considered both in weights computation and the aggregation process. Moreover, we choose to do intra-normalization of each vector first, and then concatenate them, followed by the $L_{2}$ normalization. Thus, the K·D-dimensional feature vector is generated. After the trimmed VLAD block, we use the fully connected layer to select useful spacial features for getting a *Q*-dimension compact global descriptor.

### 3.2. Image Feature Extraction

As depicted in Section 1, the visual feature extraction is very important for place recognition, since vision is the main source of obtaining information for mankind. Here we choose the ResNet50 [27] for the following two reasons. Firstly, the images collected by a camera in outdoor large-scale scenes belong to natural images. The ResNet performs well at recognizing natural images. Secondly, as a deep network, the ResNet50 has a good capability for extracting the deep features of images, and it uses a unique building block to firstly reduce the dimension of the features and then to increase the dimension. By continuously utilizing such blocks, the computation consumption is acceptable. Compared with ResNet101, the ResNet50 consumes less computation and already meets our demand, since our motivation is to verify the efficiency of our fused network. That means the additional LiDAR sensor information can improve the place recognition. The original output of the ResNet is an 1000-dimension vector, here we resize it in *Q*-dimension. The details about the image feature extractor via ResNet can be found in [27].

Here we use the ResNet as our image feature extractor rather than that followed by a NetVLAD layer. The NetVLAD, inspired by the VLAD, is a clustering algorithm utilizing the soft assignment in the neural network instead of the hard assignment. Images contain a large amount of information, and the ResNet has the capability to obtain these sufficient and deep features by its residual blocks. These appearance based features have a mutual effect with the structural point cloud features. However, arranging these features through an additional NetVLAD layer may lose a part of information. As a result, we only use the ResNet to extract image features, followed by the $L_{2}$ normalization to make the image and the point cloud components in equal weights. The comparison of fusing these two kinds of image features with the point cloud features can be found in Section 4.3.4.

### 3.3. Metric Learning for Fused Global Descriptors

As mentioned, a good mapping (or descriptor) should consist of the efficient feature extraction and the best metric to evaluate sample similarity in the fused global feature space. Different from PointNetVLAD [36], our network needs to learn how to pick the appropriate viewpoint for the fused global descriptor of image and point cloud in the fusion process (the red block in Figure 5).

To deal with the fused information, we firstly concatenate the two kinds of features, $f_{P}$ and $f_{I}$, into a long vector roughly. Then we use the fully connected layer to select useful parts in a feature vector that combines a mixture of image and point cloud features. It is worth mentioning that the global descriptor often needs to be normalized, i.e., ∥x∥=1. So, $L_{2}$ layer is conducted to balance each feature. This operation is able to eliminate the negative influence made by dimensionality. Thus, a coarse fused global descriptor $x\in R^{Q}$ is acquired.

After we get a set of descriptors *x*, the supervised label can be introduced to evaluating the similarity and dissimilarity relationship between them in the fusion global feature space. Metric is often used to evaluate the similarity of samples [56]. Meanwhile, finding the best metric is equal to finding the best mapping to a new space, where we can get the proper viewpoint to describe the data. So, metric learning is utilized to optimize the parameters in the network, i.e., it learns an appropriate mapping from the original fused descriptor $x_{i}$ to the further updated descriptor $\hat{x_{i}}$ in this module. The place recognition here maps the original input point cloud and image into a new data space where the data have the better description and discrimination.

For well fitting the data and quantifying the similarity of the fused global descriptors, we introduce a triplet constraint in an intuitive way, that is,

$$T=\{\left(x_{i},x_{j},x_{k}\right):\delta_{ij}<\delta_{ik}\},$$

where $x_{i}$ is similar to $x_{j}$, and dissimilar to $x_{k}$; $\delta_{ij}$ means the distance between $x_{i}$ and $x_{j}$, and $\delta_{ik}$ is the distance between $x_{i}$ and $x_{k}$. It means that the distance of samples in different places should be as large as possible, while as small as possible in the same place. Note that the supervised label information is needed in this triplet constraint. The traditional hinge loss function [57,58] is often used for the triplet constraint $\delta_{ij}+\alpha <\delta_{ik}$ as

(5) $$L_{H}=\sum_{T}\;\;{\left[\alpha +\delta_{ij}-\delta_{ik}\right]}_{+},$$

where ${\left[m\right]}_{+}=m$ if m≥0 and 0 otherwise, and α is the margin value to balance the inner class and intra class. So the minimization of the loss function $L_{H}$ makes $x_{i}$ closer to $x_{j}$ and maintains a margin between $x_{i}$ and $x_{k}$.

Furthermore, deep neural network based metric learning is utilized to learn an appropriate mapping M from the original fused descriptor $x_{i}$ to the further updated descriptor $\hat{x_{i}}$ in this module. The parameters of the mapping (model) are optimized via the deep network training. Similar to traditional triplet constraint based metric learning method, the descriptors in the same place should get closer and the ones in the different places get farther. Here, we want further augment this constraint, since the outdoor large-scale place recognition needs a good discrimination for the descriptors. Thus, we apply the lazy triplet constraint loss [36] to learn a discriminative mapping M.

Taking each current frame as the anchor frame $S_{anc}$ in the training dataset, its corresponding fused global descriptor is $x_{anc}$, while $x_{pos}$ represents the descriptor of place that is similar to the anchor place, $x_{neg}$ represents a dissimilar descriptor. Then, we use the descriptors of anchor frame, that of the corresponding positive and negative frames to construct a set of tuples $T=\{x_{anc},\left\{x_{pos}\right\},\left\{x_{neg}\right\}\}$.

Mathematically, the lazy triplet loss is calculated as:

(6) $$L_{lazytrip}\left(T\right)=\min\left({\left[\alpha +\sup\delta_{pos}-\inf\delta_{neg}\right]}_{+}\right),$$

where $\delta_{pos}$ is the Euclidean distance between the fused global descriptor of anchor frame and that of the one in $\left\{x_{pos}\right\}$, that is, $\delta_{pos}=\left\{d\left(x_{anc},x_{j}\right),x_{j}\in \left\{x_{pos}\right\}\right\}$; the definition of $\delta_{neg}$ is in the same way, that is, $\delta_{neg}=\left\{d\left(x_{anc},x_{k}\right),x_{k}\in \left\{x_{neg}\right\}\right\}$; $\sup\delta_{pos}$ means the supremum in $\left\{\delta_{pos}\right\}$, and $\inf\delta_{neg}$ means the infimum in $\left\{\delta_{neg}\right\}$. The sup and inf here reflect the concept of lazy in $L_{lazytrip}$. With such a loss, only the supremum in $\left\{\delta_{pos}\right\}$ and the infimum in $\left\{\delta_{neg}\right\}$ are involved in updating the parameters in model rather than the mean value of all the members.

The objective of $L_{lazytrip}$ is to minimize the supremum in $\left\{\delta_{pos}\right\}$ and maximize the infimum in $\left\{\delta_{neg}\right\}$, which is equivalent to reducing the distance between the global descriptor of $x_{anc}$ and $\left\{x_{pos}\right\}$, and extending the distance between the global descriptor of $x_{anc}$ and $\left\{x_{neg}\right\}$. With the closest negative distance and the farthest positive distance, the parameters can be updated efficiently. After using the metric learning to update the parameters in the network, $\hat{x}$ gets more discriminative.

Finally, having a trained model, we can build the desired descriptors of all the places in the dataset. Once searching an unknown place, we can map the source data *S* to the space X and then query its descriptor *x* in the database to find the candidates in a relatively small range.

## 4. Experiments and Results

To make a fair comparison with different methods, we compare our approach with the existing open source algorithm NetVLAD [34] and PointNetVLAD [36] on the same device. The device is equipped with a Tesla P40 GPU with 24GB memory, and is implemented with Ubuntu16.04 operating system carried with TensorFlow. For the super parameters of our network, we set the number of original cluster size and non-informative cluster size as 64 and 4 respectively, and margin α=0.8. The dimension of output descriptor in every part is uniformly set as 256. The training process of our method takes 40 h.

### 4.1. Datasets and Pre-Processing

We choose two large-scale outdoor datasets KITTI (Karlsruhe Institute of Technology and Toyota Technological Institute) [59] and KAIST (Korea Advanced Institute of Science and Technology) [60] for experiments. The scenarios of KITTI dataset are diverse, capturing real-world traffic situations and ranging from freeways over rural areas to urban scenes with many static and dynamic objects. KAIST dataset is composed of complex urban scenes. These two datasets satisfy our demand that the frequencies of camera and LiDAR information are the same or multipled, which is able to make the pair of image and point cloud in the same place. KITTI dataset supplies 11 scenes containing accurate odometry ground truth information. These 11 scenes are written as KITTI 00, ⋯, KITTI 10, and then we utilize these scenes for our experiments. Each scene in the KAIST dataset has accurate GPS information, so we focus on 5 scenes, where most of them have loops for place recognition evaluation.

#### 4.1.1. Kitti Dataset

KITTI Odometry split consists of 22 scenes that the images and point clouds strictly match. Half of the sequences have the ground truth poses of each frame. We only use these 11 scenes with the closed loops in our experiment.

**Figure 7:**
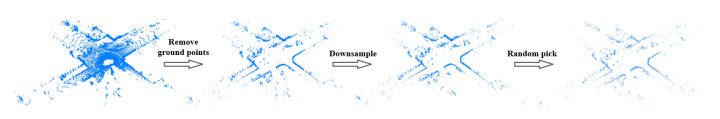
The point cloud pre-processing in the KITTI dataset.

As mentioned in Section 3, the quantity of points in each point cloud is not the same, so we need to resample the points in a fixed number. Figure 7 shows the pre-processing of the point cloud in the KITTI dataset. After checking the sample point cloud of HDL-64 LiDAR, we consider that the ground points are redundant to form a discriminative descriptor utilizing the structure information of each frame. Besides, removing these ground points can reduce the consumption of the memory. As a result, the ground points are removed using the method in [61]. Then the point cloud is downsampled into a certain quantity. Here we use the downsample API in the PCL Library, and the leafsize which represents the size of grid cells in the point cloud is set to (0.3,0.3,0.1). Finally, we randomly pick N=6000 points as our input of LiDAR to give a slight disturbance to the point cloud. For the image part, considering the size of raw input, we resize them into 180×600 on all the sequences. We choose such a large dimension of input to keep more information of raw data, while the network can also be trainable on a single GPU.

#### 4.1.2. Kaist Dataset

**Figure 8:**
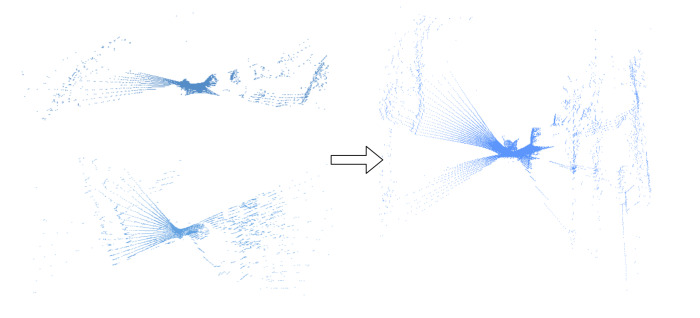
Merging the left and right point clouds in KAIST dataset. The top point cloud is from the right LiDAR, and the bottom one is from the left LiDAR.

The KAIST dataset mainly focuses on the urban scene. Images, point clouds and GPS information are acquired in different frequencies in the dataset, but the timestamps are available for all the sensors. We firstly generate data tuples depending on the timestamp of each frame among the different sensors. The difference on time of data tuple is under 0.1 s. After getting sequential frames like the data format in KITTI dataset, KAIST dataset can be operated in similar way. Notice that the images in KAIST dataset are in 8-bit Bayer format, so we demosaic them to recover the raw data into RGB images. Besides, the point cloud in KAIST consists of two VLP-16 LiDARs. We use the extrinsics between sensors and carrier to transform these two point clouds in carrier coordinate system. The merging process is shown in Figure 8.

#### 4.1.3. Our Campus Data

**Figure 9:**
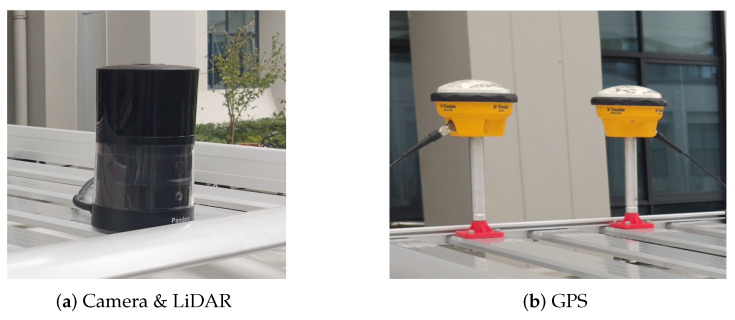
The devices used to acquire the campus data.

The hardware platform is constructed from the HESAI Pandora and the Trimble GPS in Figure 9. The Pandora contains a set of sensors, which are 4 greyscale cameras, a color camera and a 3D LiDAR. While the GPS consists of a receiver (SPS461) and 2 antennas (GA530). Here we use the GPS data as ground truth. The LiDAR in Pandora has 40 scanners so that we can get a dense point cloud of surroundings. As a result, our campus data can use the same pre-processing in KITTI dataset. The related experiments can be found in Section 4.3.6.

#### 4.1.4. Triplet Tuple

We use the ground truth trajectory to generate training tuples. Every tuple is composed of two parts, that is, image and point cloud, respectively. Both of them contain the keys $S_{anc}$, $\left\{S_{pos}\right\}$ and $\left\{S_{neg}\right\}$. For each anchor frame $S_{anc}$, $\left\{S_{pos}\right\}$ is generated in terms of position distance and the time. The distance between the candidate positive frame $S_{pos}\in \left\{S_{pos}\right\}$ and $S_{anc}$ is under 5 m and the time difference is under 10 s. Similarly, the distance between candidate negative frame $S_{neg}$ and $S_{anc}$ is over 50 m. Through random selection from these candidate frames, the input training tuple can be finally generated. This operation also increases the robustness of the trained models. Additionally, we take the frames passing through the same place as the evaluation frames.

### 4.2. Place Recognition Results

Here we evaluate our approach to the KITTI and KAIST datasets. To the best of our knowledge, few methods combine both the image and the point cloud on the place recognition task, so we compare the proposed approach with some methods on a single sensor mentioned before. The NetVLAD is an image-based method which utilizes the concept of VLAD to develop a unique layer which aggregates the image features extracted by a CNN architecture. Here we use the ResNet50 to extract visual information. Besides, the output of ResNet is a vector which can also be seen as a compact representation of an image. Therefore, the ResNet is also be included. The PointNetVLAD is a point-based approach combining the concept of PointNet and NetVLAD, which applies an image-based method to the point cloud. The PointNetVLAD with the trimmed strategy will also be included. For a fair comparison, we use the same training datasets to train the models and then do the retrieval.

**Figure 10:**
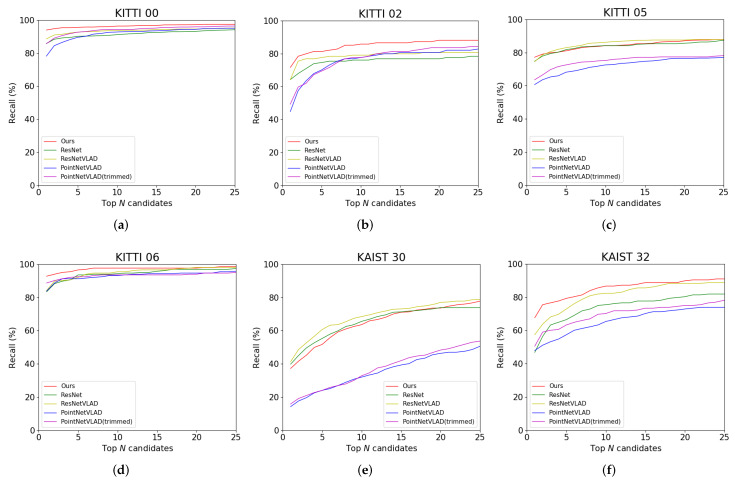
Comparison of our approach with other approaches on KITTI and KAIST datasets.The ResNet and ResNetVLAD are image-based approaches. Both of them use the ResNet as the feature extractor, and the only difference is the final layer. The PointNetVLAD and PointNetVLAD (trimmed) are point-based approaches. PointNetVLAD is open source, and the trimmed version uses our non-informative clusters.

The results are given in Figure 10 and Table 1. As mentioned in Section 1, the LiDAR and camera data have mutual effect on each other. Intuitively, when both the image and the point cloud parts have a good ability to distinguish the similar and dissimilar places, fusing these two features will have a better performance. Because of the lower recall rate on the LiDAR-only descriptor, in the curves of KITTI 05 and KAIST 30, the performance of our fusion feature is worse than the image-only method in the certain segment. But combining the visual information with point cloud information reduces the bad discrimination of the LiDAR-only descriptor. It can also be observed in Table 1. Although the frames in each scene are different, the results of the top 1% of candidates, as our benchmark, show the average performance of all the approaches.

In the KAIST dataset, because the point cloud only contains a part of the structure of the whole scene, the results of LiDAR are much lower than that of the image. Generally speaking, our fusion approach is more accurate and robust.

### 4.3. Analysis and Discussion

In this subsection, we discuss the results of our network in detail. We determine the parameters, show the impact of the non-informative clusters, compare the different image features extractor mentioned before, further demonstrate the advantages of our fusion descriptor and analyze the utility of our method.

#### 4.3.1. Number of Points

In Table 2, we firstly discuss the quantity of the points in each point cloud *P*. The more points retained after downsampling from the original point cloud, a better the performance of model is. However, the more points used in the network, the more computing resources are occupied. As a compromise, we set a fixed number of points N=6000 in our method.

#### 4.3.2. Number of Cluster Centers

Table 3 shows the recall of top 1 candidate in different number of cluster centers K. Intuitively, this parameter is due to the points number *N* and the complexity of the scene. The more points or the more complex the scenes are, the more cluster centers is required. In our experiments, K=64 achieves a better performance.

#### 4.3.3. Effect of the Non-Informative Clusters

**Figure 11:**
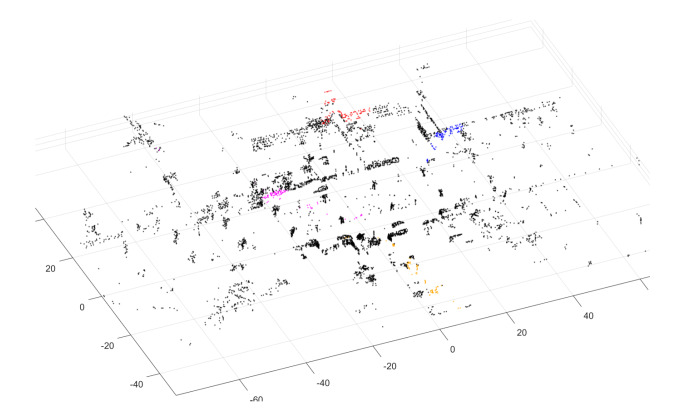
Visualization of non-informative clusters. The marked points in the same color belong to the same non-informative cluster. For each non-informative cluster, most of the points are gathered together, but some points are far away from the center.

Here we use the recall rate of top *N*-number candidates in the database to examine whether the non-informative clusters can improve the PointNetVLAD. Figure 10 and Table 1 show the comparison of PointNetVLAD and our trimmed version. In conclusion, our trimmed strategy performs better than the original PointNetVLAD. Meanwhile, we rank the weights of the points to all the clusters. The cluster with the biggest weight is defined as the owner of the point. Then we marked out the points belonging to the non-informative clusters with the different colors in Figure 11. It is shown that the non-informative clusters are not so meaningful. Specifically, these points of abandoned clusters in our trimmed approach contain the outliers or isolated points. Therefore, the concept of trimmed strategy makes network learn how to ignore these non-informative clusters, which helps to improve the quality of features computed by the trimmed VLAD block.

#### 4.3.4. Different Image Features

We fuse two different image features with the point cloud part in our fusion network. One is the features directly extracted from the ResNet50, and the other is the features extracted from the ResNet50 and followed by a NetVLAD layer. The ResNet is a deep network with stacked residual blocks. Its final layer is a fully connected layer, which has already downsampled the features. To some extent, it is also a concept of local aggregation. Therefore, in our fusion network, fusing the features from ResNet without the NetVLAD layer with the point cloud features is reasonable (Table 4).

While the point features are orderless and there are only a few methods designed on the point cloud, using the VLAD here can efficiently aggregate the point features to a global point cloud feature. In Table 4, we use the KITTI dataset to further verify our observation. Here we show the result of the top 1% of candidates and the top 1 candidate to compare the effect of fusing these two image features with the spatial feature. In our fusion architecture, ResNet without NetVLAD layer has a better performance in overall cases.

#### 4.3.5. Effect of Learned Descriptors

**Figure 12:**
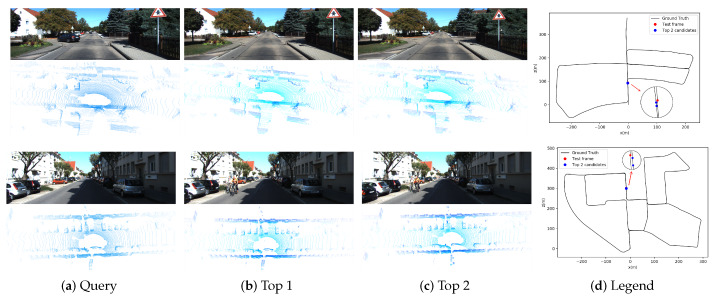
Top 2 candidates of two scenes in the KITTI dataset. The position of the test frame and the top 2 candidates are marked on the trajectory. The candidates of each test frame are more likely to be adjacent frames.

Figure 12 shows the top two candidates of two test frames using our fusion method. Our method can still find the corresponding frames in database, even there are some moving objects in datasets. Besides the top candidates of test frame in database are usually adjacent frames, our fused descriptor has a good performance on retrieval.

**Figure 13:**
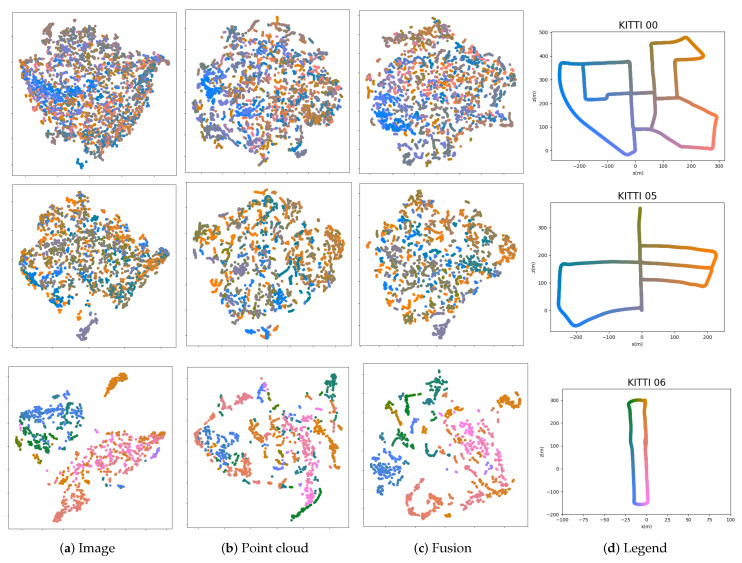
t-SNE plot of several scenarios in image, point cloud and fusion methods. The positions of the frames are represented by each point in a certain scene, where the adjacent relationship is represented by its color. We use the ground truth in the KITTI dataset to set all frames in a specific color, which means the adjacent frames have the similar colors.

**Figure 14:**
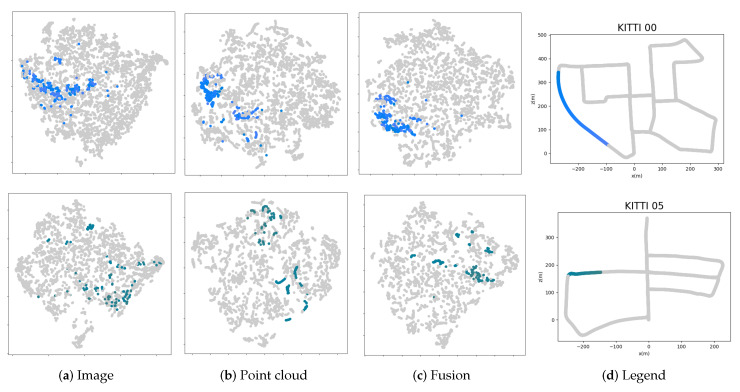
Illustration of t-SNE plots. We highlight a short segment on the trajectory and mark the corresponding points in the plots.

Here we show the visualization of the features in several scenes. The numbers of frames in each scene are in different scales. In Figure 13, we use the t-SNE to represent three kinds of global descriptors—image, point cloud and fusion, which are generated from the ResNet, PointNetVLAD and our fusion way respectively. In addition, we also draw the legend with gradient colors in each scene. In the t-SNE plot, the more points in different colors are mixed, the worse the discrimination of the descriptors is. In other words, the adjacent frames are similar intuitively, so the corresponding points in the plots should be closer. From this perspective, our method has fewer mixed areas in overall cases. In Figure 14, we select short segments to explain the t-SNE plot in detail. Note that the marked area on the trajectory corresponds to the frames represented by the points in the plots. In our fusion method, there are less isolated points than those in other methods. It means our descriptor has a better performance in the feature space.

#### 4.3.6. Usability

Finally, we further study the application of our method. In Table 5, we list the recall and the average processing time between the VLAD and our method. Here we use the ORB feature to detect the corners and aggregate the hand-crafted features in VLAD vector. Our method performs better in overall scenes. Note that the VLAD method needs to calculate the local descriptors in the image before aggregation, so the processing time here also includes the time to extract features and corresponding local descriptors. While our method can directly get a global descriptor from the model. Less time is needed in our approach to form a descriptor. Compared with the VLAD algorithm, which has been proven to be practical, our method has the potential to be applied in the field of autonomous driving or robotics systems.

**Figure 15:**
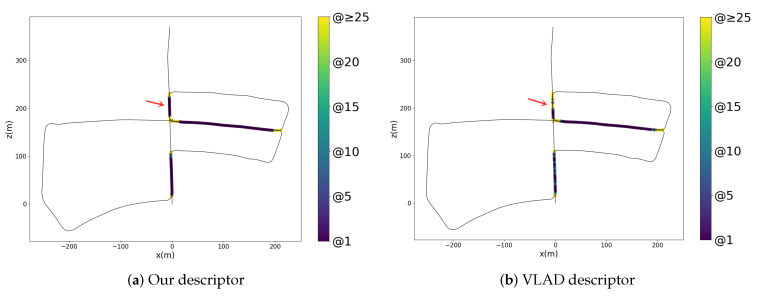
The retrieval map of KITTI 05 in our approach and the VLAD algorithm. Here we mark the minimal required candidates that contain the correct correspondence in the whole trajectory.

Besides, we use the heat map to visualize the minimal required candidates of finding the corresponding frame in database. In Figure 15, those tested frames are marked on the trajectory by specific colors. The color represents the least required candidates to find the corresponding frame. In our method, most of the marks on the map are dark, and only a few points near the intersection are bright. Compared with the hand-crafted method, our method is also capable of the place recognition task.

**Figure 16:**
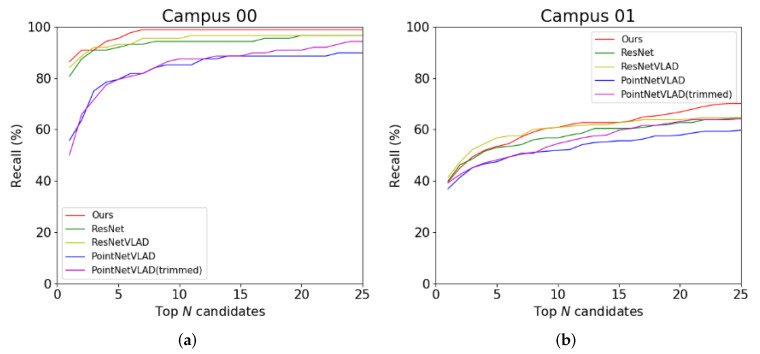
Comparison of our approach with other approaches to our campus dataset.

Here we also test the proposed approach in our campus. In Figure 16, we show the comparison against the state-of-the-art methods in our campus, the results on the sensors rather than the public datasets show our fusion method is competitive.

## 5. Conclusions

In this paper, we have proposed a novel network for place recognition by fusing the information from the image and the point cloud. We apply the trimmed strategy in the point cloud global feature aggregation to avoid the perturbation of complex environments. Moreover, we fuse this compact global descriptor of the point cloud with that of the corresponding image to get a robust fused global descriptor for each place. Finally, we learn a proper metric to describe the similarity of our fused global feature to get an end-to-end place representation network. We implement our approach and some off-the-shelf methods on the open source KITTI and KAIST datasets. Experiments and visualization show that the fusion of two kinds of sensors can improve the performance, that is, the descriptor generated from two sensors is more robust than that from a single sensor.

## Figures and Tables

**Table 1:** Comparison against the state-of-the-art approaches (@1%). The symbol @1% means recall (%) on the top 1% candidates.

	ResNet	ResNetVLAD	PointNetVLAD	PointNetVLAD (trimmed)	Ours
KITTI 00	95.6	96.3	96.4	97.6	98.1
KITTI 02	80.6	83.6	84.3	84.3	88.1
KITTI 05	87.6	87.9	78.2	78.6	88.1
KITTI 06	94.7	95.5	93.6	93.6	97.7
KAIST 30	84.2	83.7	72.6	72.4	87.3
KAIST 32	88.3	92.6	86.7	93.1	95.2

**Table 2:** Comparison of differernt numbers of points (@1). The results are tested on the KITTI dataset. The symbols @1 means recall (%) on the top 1 candidate.

	N=1024	N=2048	N=4096	N=6000
KITTI 00	57.5	71.7	76.1	76.6
KITTI 02	21.6	34.3	35.1	43.3
KITTI 05	41.0	52.7	56.3	58.5
KITTI 06	50.6	70.2	80.0	83.0

**Table 3:** Comparison of differernt numbers of cluser centers (@1). The results are tested on the KITTI dataset. The symbols @1 means recall (%) on the top 1 candidate.

	K=32	K=64	K=96	K=128
KITTI 00	78.0	78.9	78.8	76.6
KITTI 02	38.8	45.5	46.3	43.3
KITTI 05	59.7	61.7	60.9	58.5
KITTI 06	81.5	83.4	82.3	83.0

**Table 4:** Comparison of fusing different image features (@1%/@1). The results are tested on the KITTI dataset. The symbols @1% and @1 mean recall (%) on the top 1% and 1 candidate(s), respectively.

	@1%		@1	
	ResNet	ResNetVLAD	ResNet	ResNetVLAD
KITTI 00	98.1	97.4	93.1	87.3
KITTI 02	88.1	86.6	73.9	54.5
KITTI 05	88.1	76.9	76.5	63.6
KITTI 06	97.7	95.5	94.7	86.8

**Table 5:** Comparison of the VLAD and our approach. Here we list recall(%) @1 in the KITTI dataset and processing time of extracting the global descriptor of each frame in seconds.

	@1		Time	
	VLAD	Ours	VLAD	Ours
KITTI 00	88.6	92.7	0.046	0.025
KITTI 02	63.4	64.9		
KITTI 05	66.0	76.9		
KITTI 06	82.3	92.5		
